# Long-term results of radiotherapy in anaplastic thyroid cancer

**DOI:** 10.1186/1748-717X-9-90

**Published:** 2014-03-31

**Authors:** Anne-Katrin Dumke, Tanja Pelz, Dirk Vordermark

**Affiliations:** 1 Department of Radiation Oncology, Martin Luther University Halle-Wittenberg, Dryanderstr. 4, 06110 Halle (Saale), Germany

**Keywords:** Anaplastic thyroid cancer (ATC), Undifferentiated thyroid cancer (UTC), Radiotherapy, Multimodal therapy, Prognostic factors, Long-term survival

## Abstract

**Background:**

Anaplastic thyroid cancer (ATC) is an aggressive malignant tumour with a poor prognosis. The median overall survival is described in the literature to be just 6 months, however, in series of selected patients treated by multimodal therapy cases of long-time-survival have been reported. We analyzed the role of radiotherapy and the impact of other therapies and clinical features on survival in patients with ATC.

**Methods:**

In a retrospective analysis of all patients (n = 40), who presented with histologically proven ATC at a single centre between 1989 and 2008, patient and treatment characteristics with a focus on details of radiotherapy were registered and the survival status determined.

**Results:**

39 of 40 patients received radiotherapy, 80% underwent surgery and 15% had chemotherapy. The median dosis of radiation was 50 Gy (6–60.4 Gy), in 87.5% fractionation was once daily. In 49.4% opposing-field techniques were applied, in 14% 3D-conformal-techniques and 32.5% combinations of both.

The median overall survival (OS) was 5 months, 1-year survival 35.2% and 5-year-survival 21.6%. Interestingly, 24.3% survived 2 years or longer. Three factors could be identified as predictors of improved overall survival: absence of lymph node metastasis (N0) (median OS 18.3 months), median dose of radiation of 50 Gy or more (median OS 10.5 months) and the use of any surgery (median OS 10.5 months).

**Conclusions:**

Despite the generally poor outcome, the combination of surgery and intensive radiotherapy can result in long-term survival in selected patients with ATC.

## Background

Anaplastic thyroid cancer (ATC) is a highly malignant tumor originating from follicle cells with a peak incidence in the sixth to the seventh decade and preponderance in women [1,2]. The WHO classification of thyroid cancer distinguishes between the well-differentiated thyroid carcinoma (WDTC) - papillary and follicular - and undifferentiated/anaplastic thyroid carcinoma (UTC, ATC). Poorly differentiated thyroid carcinoma (PDTC), has been defined as a histologic type inbetween these two groups [3].

ATC is known as one of the most serious malignant tumors with a median overall survival of 6 months or less [2-4]. It accounts for less than 5% of all thyroid cancer but contributes up to 90% of thyroid cancer mortality [3]. Each year, only about 420–900 new cases in Europe and 700–1200 new ATC patients in the USA are observed [5].

Patients usually present with a rapidly enlarging mass and symptoms like stridor, dysphagia, vocal cord paralysis, neck pain and dyspnea. Additionally in about half of ATC patients there is evidence of metastatic disease at initial diagnosis [1], the most common sites being lung, brain and bone [6]. At the end of follow-up in most published reports, the majority of patients have developed distant metastasis [1,7].

The etiology of ATC is heterogeneous. It has been found out, that genetic components as well as a lack of iodine, radiation, female sex and nicotine abuses are important factors. Recent molecular pathological studies elucidated the activation of various tyrosine cascades and inactivation of the TP53 tumor suppressor gene. ATC occasionally develops from previous WDTC or a long standing goiter [1,2,8].

As outlined in the recent American Thyroid Association guidelines for the management of patients with anaplastic thyroid cancer, there are so far no data from randomized trials of ATC treatment available [9]. Treatment recommendations must therefore be based on the experience from case series, non-randomized trials and epidemiologic databases. We, therefore, performed an analysis of long-term results of radiotherapy in ATC patients treated at a single center.

## Methods

We performed a retrospective analysis of all 40 patients (26 female, 14 male) who presented with histologically proven ATC at the Dept. of Radiation Oncology, Martin Luther University Halle-Wittenberg, Germany between 1989 and 2008. Information on patient and treatment characteristics was systematically collected from the patients’ records. Survival status was determined for each patient via local registry offices. This retrospective analysis was performed in compliance with the Helsinki Declaration.

The survival analysis was implemented by the software Statistica (Version 10, Fa. Stat. Soft, Tulsa, OK, USA) and used the Kaplan-Meier method. The endpoint in this study was overall survival, where death of any cause was considered an event. Patients alive at last follow-up were censored. Overall survival for the whole cohort and in subgroups defined by clinical and therapeutic factors was calculated by the Kaplan-Meier method and compared between subgroups using the log-rank test (p < 0.05 considered significant).

## Results and discussion

Patient characteristics are presented in Table 1. In the present cohort 65% of patients were female, the age range was 38–84 years (median age 67). NO, N1 and N3 status were present in 23%, 40%, 3% (remainder Nx). Distant metastases were reported in half of all cases, 25% at diagnosis, and another 25% in the course of treatment (32.5% lung, 7.5% bone, 5% liver, 2.5% skin, 2.5% jejunum, 2.5% mediastinum and 2.5% brain; multiple site possible). In view of the long observation period each tumour stage was assigned to the TNM classification valid at the time of treatment (UICC 5^th^ edition 1997 and 6^th^ edition 2002). If the current TNM classification (UICC 7^th^ edition 2009) is taken as a basis, a difference in lymph node status results in 5 cases [10]. However, all ATC patients are stage IV in all versions.

**Table 1:** Patients baseline characteristics (n = 40)

**Age (in years/range)**	38-84
**Sex**	
♀	65%
♂	35%
**N stage**	
N0	23%
N1	39%
N3	3%
**Distant metastasis**	
At diagnosis	22.5%
In the course of treatment	27.5%
**Surgery**	
Total thyroidectomy	68%
Subtotal thyroidectomy	12%
**Resection status**	
R0	10%
R1	50%
R2	7.5%
Rx	32.5%
Removal of lymph nodes	32.5%
**Chemotherapy**	15%
**Radiotherapy:**	97.5%
Opposing field technique	49.5%
3-d-conformal technique	14%
Combination of both	32.5%
Period between operation and radiation (in days/range)	13 (2–399)
Median applied dose (Gy/range)	50 (6–60.4)
**Fractionation schedules**	
Daily radiation	87.5%
Twice daily radiation	7.5%
Radiation every second day	7.5%

After surgery which was accomplished in 80% of cases (68% total, 12% subtotal thyroidectomy), only 10% of those treated with surgery achieved a microscopic complete resedction (R0). 50% had R2 status, 7.5% R1 status. In one third of patients the resection status could not be determined. In 50%, a removal of lymph nodes was performed.

Six of 40 patients (15%) received any documented chemotherapy, in three cases only before radiotherapy, in one case only during radiotherapy, in one case only after radiotherapy and in one case before, concomitant with and following radiotherapy. The protocols used were adriblastin monotherapy (two patients), paclitaxel monotherapy, combination ifosfamide/carboplatin/etoposide, epirubicin monotherapy and unknown protocol (one patient each). It appears possible that further palliative chemotherapy may have been given in some cases after radiotherapy in outside institutions. In combination with surgery, radiotherapy was always applied postoperatively.

Radiotherapy was delivered in 39 cases (one patient refused radiotherapy). Various irradiation techniques were applied: in 49.5% opposing-field technique only, in 14% 3D-conformal technique and in 32.5% cases a combination of both. Median total dose was 50 Gy (range 6 to 60.4 Gy). In 87.5% radiotherapy was given once daily, in 5% every other day and in 5% twice daily. In 8 patients radiotherapy could not be completed because of deterioration of general condition.

**Figure 1:**
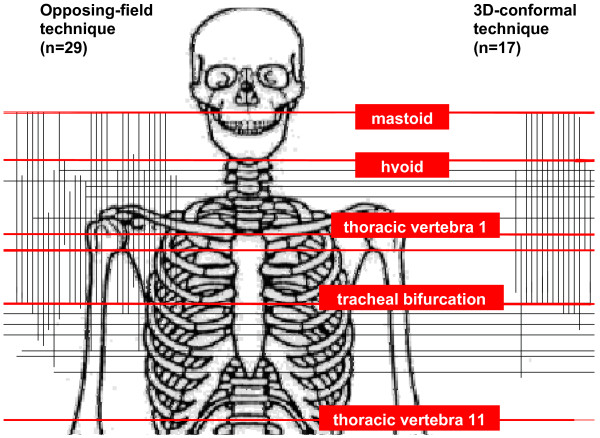
Overview of anatomic positions of upper and lower field borders as used in opposing-field techniques and 3D-conformal techniques for anaplastic thyroid cancer (combinations of both techniques used in some patients).

Target volume concepts varied. The most frequent combination of anatomic locations for upper and lower border, mastoid to tracheal bifurcation (+/−2 centimetres), was applied in 27.6% of opposing-field techniques and 47% of 3D-conformal techniques. The distribution of upper and lower field borders in relation to anatomic landmarks is displayed in Figure 1.

CTCAE (version 3.0) skin toxicity was reported as grade 1, 2, or 3 in 28%, 45% and 13%, respectively. Grade 1, 2, 3 or 4 dysphagia was observed in 35%, 28%, 25% and 3%, indicating that 28% of patients required tube feeding or parenteral nutrition. During the course of treatment, 7 patients (18%) underwent emergency tracheotomy and in 47.5% had recurrent laryngeal nerve palsy. During treatment 8 patients (20%) suffered from acute dyspnea attacks, one patient suffocated because of shortness of breath and blood loss from the tracheostomy wound.

**Figure 2:**
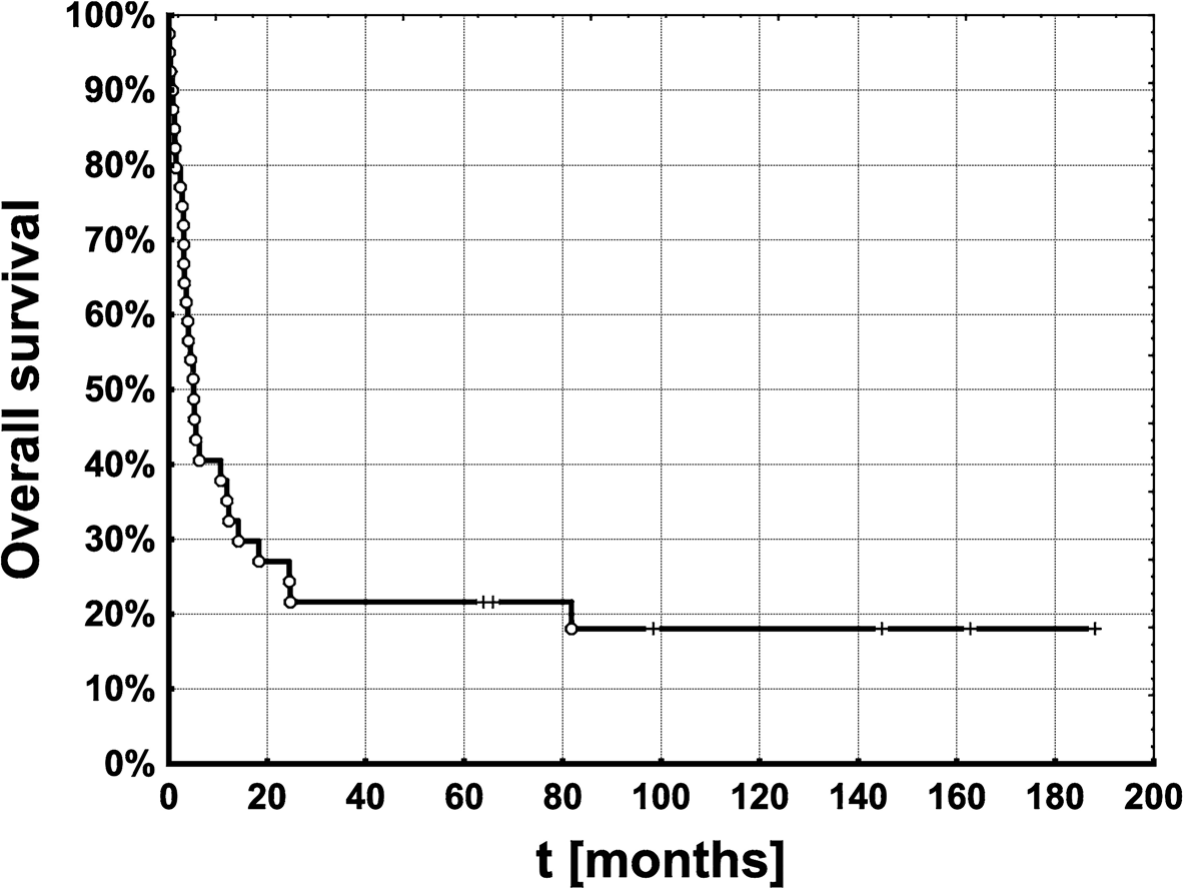
Overall survival of n = 40 patients with histologically proven anaplastic thyroid cancer.

**Figure 3:**
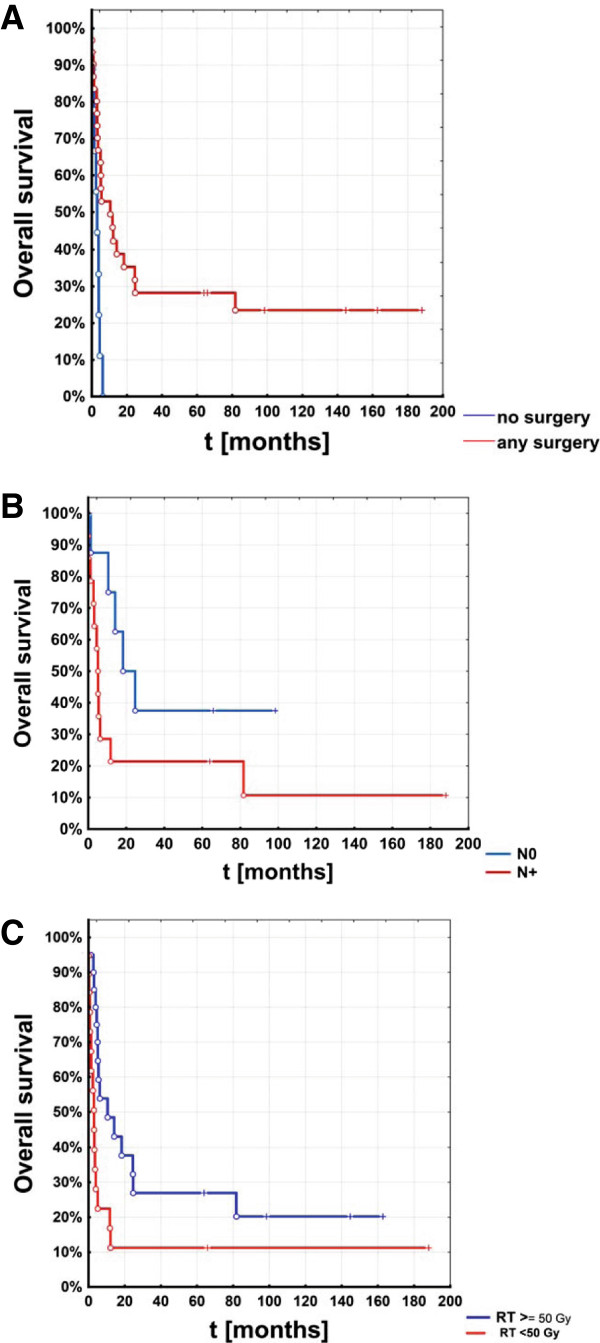
Overall survival in subgroups of patients with anaplastic thyroid cancer: significantly improved survival was seen in (A) patients with any surgery vs. no surgery (p = 0.02) (B) patients with N0 status vs. N + status (p = 0.041) and (C) patients with a radiotherapy dose of at least 50 Gy vs. lower doses (p = 0.022).

The median overall survival of all 40 patients was 5 months, 43.3% were alive at 6 months and 35.2% at one year. Interestingly, 21.6% of patients survived 5 years or longer after first presentation (Table 2, Figure 2). Three significant factors were found to be predictors of higher overall survival: absence of lymph node metastasis (N0), median dose of radiation of 50 Gy or more and use of surgery. Six months after presentation patients with surgery had a median survival rate of 52.9%, in the comparison group however it was just 11.1%. Median survival of patients who underwent a surgical intervention was 10.5 months vs. only 3 months without (p = 0.02, log-rank test) (Figure 3A). A selection effect may be considered since only patients in sufficient general condition were candidates for surgery.

**Table 2:** Survival of anaplastic thyroid cancer patients in subgroups

**Parameter**	**Subgroups**	**Median overall survival (months)**	**p**
All patients		5	
Surgery	Yes	10.5	0.02
	No	3	
N Stage	N0	18.3	0.041
	N+	4.5	
M Stage	M0	18.3	0.07
	M1	4	
Age	<67 years	5.5	0.39
	≥67 years	4	
Sex	♀	5	0.66
	♂	5	
Histology	“Pure ATC”	4.5	0.29
	“ATC with PDTC”	10.5	
Total RT dose (Gy)	≥50	10.5	0.02
	<50	3	
Resection status	R2	3.6	0.41
	R0, R1	12.2	
Lymph node dissection	Yes	12.2	0.051
	No	3	
Chemotherapy	Yes	4	0.85
	No	5.5	

ATC patients without lymph node metastases (N0) had a median survival of 18.3 months (Figure 3B); in the comparison group it was significantly lower (4.5 months). 1-year and 5-year survival in the N0-collective was 75% and 37.5%, patients with lymph node metastases had a cumulative survival of 18.8% at both timepoints (p = 0.041).

ATC patients who received a total dose of 50 Gy or more had a median survival of 10.5 months, whereas the comparative group only survived 3 months. Cumulative survival among patients irradiated to the higher doses was 59.2%, 46.5% and 26.9% after 6 months, 1 year and 5 years, respectively; in the control group merely 22.5%, 16.8% and 11.2% (Figure 3C).

Non-significant trends toward improved survival were observed for surgical removal of lymph nodes (p = 0.051) and absence of distant metastasis (p = 0.07). No effects were seen for resection status or use of chemotherapy.

We compared the nine patients with survival until last follow-up with the rest of the cohort to obtain additional information on potential prognostic factors. As expected they were younger (median age: 64 vs. 69 years), less often had lymph node metastasis (44.4% vs. 16.1%) and reached R0 status more frequently (22.2% vs. 6.5%). Median radiation doses were not different (50 vs. 48 Gy). The presence of nine long-term survivors after treatment of ATC in was an unexpectedly favorable result. It raised the question, if all patients actually had ATC. Review of histologic reports clearly confirmed that all patients were diagnosed and treated as ATC. In view of recent changes in histologic classification, we considered that some patients previously classified as ATC might be grouped as poorly differentiated thyroid cancer (PDTC) in the current classification. The presence of ATC within areas of tumor types which would now be classified as PDTC (e.g. insular carcinoma) was described in seven patients, whereas in the remainder only ATC was described. The median survival was 10.5 months in patients with “ATC with PDTC” vs. 4.5 months in patients with “pure ATC” (p = 0.29), suggesting that – although all cases were clearly identifiably as ATC and although the difference is not significant – histologic subtypes may have contributed to differences in survival.

Results of radiotherapy or multimodal therapy of ATC have mostly been reported from retrospective studies [11,12]. Though aggressive radiotherapy could reduce locoregional recurrences, median overall survival did not improve during the last 50 years [13]. In almost all analyses it was realized that only a multimodal therapy consisting on surgery, radio- and possibly chemotherapy is advisable since just one modality rarely leads to tumor control, with combinations producing increased toxicity [11,14]. Local control seems to be an important factor for overall survival [11]. According to Levendag et al. [15] who analyzed 51 patients treated over a period of 25 years median survival under local control was 7.5 months, in the group without local control just 1.6 months. In a current study by Derbel et al., of 35 ATC patients who received three-phase-combined treatment complete response after treatment was achieved in 14/44 patients (31.8%), 8 had partial response (18.2%) and 22 (50%) had progressive disease [11].

In our collective as well as in other surveys more female patients were affected (65% of cohort). The age structure was similar to other studies, as ATC is a predominantly a malignancy of the elderly. According an American review of ATC [1], about 40% of patients at diagnosis had developed cervical lymph node metastases, which is synonymous with significantly worse survival prognosis. This perception corresponds to the proportion of lymph node metastases in the present cohort (39%).

### Role of radiotherapy in ATC

In a Canadian study by Wang [16] there were 47 patients and two therapy arms: Those with poor general condition or distant metastasis were irradiated to a total dose of less than 40 Gy (most often 20 Gy within one week), patients with good general condition received radical therapy with 40 Gy or more (median 60 Gy). In the majority of cases opposing-field technique was applied. Persons treated radically received 60 Gy in 30 fractions once daily within 6 weeks or in 40 fractions twice daily within 4 weeks. Overall survival of patients irradiated with the higher dosage was 79.8% after 6 months, 46.1% after 1 year and 9.2% after 2 years. In the group with palliative treatment, no patient survived longer than 9 months, 6-month survival amounted to 16.7%. In this study, overall survival among patients irradiated with 40 Gy or more was significantly higher than in the comparison group: median 11.1 vs. 3.2 months (p < 0.0001) [16].

In a Swedish trial by Tennvall et al. [17] there was strong correlation between accelerated radiotherapy and tumor control. Patients who were irradiated twice a day with 1.6 Gy achieved local tumor control in 77.3%. However, overall survival was disappointing (just 2 months versus 4.5 and 3.5 months). In the survey of Wang et al., median overall survival of patients irradiated twice daily was higher (13.6 months) than of those irradiated once a day (10.3 months). These results were not significant, therefore the conclusion of hyperfractionation radiotherapy having benefit on survival is considered cautiously by the authors of this survey [16]. In a recent survey by Lim et al. in a collective of 13 ATC patients the median overall survival was longer with radical radiotherapy (11.1 months) compared with palliative radiotherapy (3.2 months) [6].

Side effects of accelerated therapy and higher total dosage must also be considered. In the present study, skin toxicity and dysphagia of at least CTCAE grade 2 were each observed in more than half of the patients. Planned total dose could not be achieved in eight cases. According to a current review best success was achieved with a dosage of at least 46 Gy [1]. This perception was confirmed in the present study, as a total dose of 50 Gy or more was a positive, statistically significant prognostic factor for survival.

### Role of surgery in ATC

Prophylactic tracheostomy has been identified as a controversial factor by various authors. According to Wallin et al. local tumour growth could be promoted by tracheostomy and the start of radiotherapy might be delayed by wound healing [14]. The percentage of distant metastasis is high as well. According to Are et al. [1], 46% of patients are affected at start of treatment, in the course of disease the proportion increases to 68% (this cohort: 22.5% and 50%, respectively). In the largest ATC collective in the literature (516 patients of 12 population-based cancer registries of the USA, Kebebew et al. [18]) altogether 43% had distant metastasis. Common sites of metastasis are lung (up to 80%), bones (6-15%) and brain (5-13%) [6]. In 1996 Koboyashi et al. [19] found that by complete macroscopic tumour resection an improvement of median survival from 2 to 6 months could be achieved. A work of Junor et al. [20] with 91 patients revealed that patients who underwent a total or partial thyroidectomy with subsequent radiation survived longer than patients who had received a biopsy only. Consequently, not the performance of any surgical treatment but the extent of it is decisive.

Given the long time period of the present analysis (1989 to 2008), it is difficult to determine in individual cases how the decision on surgical management was made. As no interdisciplinary tumor board recommendation is available for the vast majority of cases, it has to be assumed that this was at the individual decision of the responsible surgeon, based on resectability, presence of distant metastases and general condition of the patient. Even in the most recent American Thyroid Association Guidelines [9] the recommendations regarding surgery are rather weak, essentially suggesting to “consider” resection both in locoregional disease as well as in systemic disease.

### Role of systemic therapy in ATC

Response rates of chemotherapeutic agents in ATC are modest, in the range of 20% for doxorubicin, bleomycin, etoposide, cisplatin and methotrexate. In a Swedish series published in 2002, accelerated, hyperfractionated radiotherapy and 20 mg doxorubicin given daily did not lead to serious side effects [17]. The impact of chemotherapy on survival is generally limited. One reason could be that according in-vitro-analyses, anaplastic cell lines form less mdr-1-mRNA and P-glycoprotein but more MRP (multidrug resistance-associated protein) which can expel cytostatic agents from cells [1]. Chemotherapy has shown beneficial effects mostly in combination with radiation.

Recently, molecular targeting strategies have been evaluated in ATC. There have been efforts to develop multikinase inhibitors to disturb signaling pathways which are involved in the formation of undifferentiated thyroid carcinoma and inhibitors of angiogenesis like combretastatin [11]. Investigations have demonstrated that the MMP-activated LeTx inhibited orthotopic ATC xenografts progression in both toxin-sensitive and toxin-resistant ATC cells via reduced endothelial cell recruitment and subsequent tumour vascularization [21]. Other systemic approach involves gefitinib which blocks the tyrosinkinase domain of the EGF receptor, which is often overexpressed in ATC-cell lines [22] and monoclonal antibodies against VEGF [23]. Other experimental studies have focussed on BMP 7 (bone morphogenic protein) [7] inhibitors of histone acetylases to augment the effect of cytostatic drugs, PPAR-γ- (peroxisome proliferator-activated receptor gamma) agonists like rosiglitazon and ciglitazone [22] and bortezomib by inhibiting the NF-κB-pathway [24].

### Concomitant radiochemotherapy

Available data on the combination of radiotherapy and chemotherapy (concomitant radiochemotherapy) are limited. In the current American Thyroid Association Guidelines [9], the following recommendation is made: “The use of cytotoxic chemotherapy involving some combination of taxane (paclitaxel or docetaxel), and/or anthracyclines (doxorubicin) and/or platin (cisplatin or carboplatin) therapy should be considered in combination with radiation therapy or altered fractionated radiotherapy in good performance status patients with nonmetastatic ATC who desire aggressive therapy.” Thus, no specific protocol for chemoradiation is recommended and the criteria for patient selection are not very clear. The recommendation is based on single-institution experiences where considerable long-term survival (in the range of 30 to 50%) was achieved in highly selected patients with locoregional disease (no distant metastases) who were treated with a trimodality regimen consisting of surgery (R0 oder R1), high-dose radiotherapy and concomitant plus sequential chemotherapy [25-27]; review in [28]. The chemotherapy regimens used in these series were heterogeneous. The examples of “adjuvant/radiosensitizing chemotherapy” listed in the 2012 American Thyroid Association Guidelines are various regimens of monotherapies or doublet combinations containing paclitaxel, cisplatin/carboplatin or doxorubicin [9]. Due to the low number of patients receiving any documented chemotherapy and especially receiving chemotherapy concomitant with radiotherapy in the cohort studied now, no information on the optimal combination can be derived from our data set.

## Conclusions

Long-term single-center results of radiotherapy for ATC indicate that despite a poor median survival of 5 months in the overall cohort, 22% of patients could be regarded as long-term survivors. A total radiation dose of >50 Gy, any surgery and the absence of lymph node metastasis were associated with improved survival.

## Competing interests

The authors declare that they have no competing interests.

## Authors’ contributions

AD was involved in conception and design, acquisition, analysis and interpretation of data and drafted the manuscript. TP made substantial contributions to conception of the project, acquisition and analysis of data and treated patients. DV contributed to conception, design, statistical analysis and interpretation of data and revised the manuscript. All authors have given final approval of the manuscript.
